# Glucocorticoids and Type 2 Diabetes: From Physiology to Pathology

**DOI:** 10.1155/2012/525093

**Published:** 2012-12-18

**Authors:** Guido Di Dalmazi, Uberto Pagotto, Renato Pasquali, Valentina Vicennati

**Affiliations:** Division of Endocrinology, Department of Medical and Surgical Science, S. Orsola-Malpighi Hospital, University Alma Mater Studiorum, Via Massarenti 9, 40138 Bologna, Italy

## Abstract

Type 2 diabetes mellitus is the result of interaction between genetic and environmental factors, leading to heterogeneous and progressive pancreatic **β**-cell dysfunction. Overweight and obesity are major contributors to the development of insulin resistance and impaired glucose tolerance. The inability of **β** cells to secrete enough insulin produces type 2 diabetes. Abnormalities in other hormones such as reduced secretion of the incretin glucagon-like peptide 1 (GLP-1), hyperglucagonemia, and raised concentrations of other counterregulatory hormones also contribute to insulin resistance, reduced insulin secretion, and hyperglycaemia in type 2 diabetes. Clinical-overt and experimental cortisol excess is associated with profound metabolic disturbances of intermediate metabolism resulting in abdominal obesity, insulin resistance, and low HDL-cholesterol levels, which can lead to diabetes. It was therefore suggested that subtle abnormalities in cortisol secretion and action are one of the missing links between insulin resistance and other features of the metabolic syndrome. The aim of this paper is to address the role of glucocorticoids on glucose homeostasis and to explain the relationship between hypercortisolism and type 2 diabetes.

## 1. Introduction 

Type 2 diabetes mellitus is a complex endocrine and metabolic disorder. The interaction between several genetic and environmental factors results in a heterogeneous and progressive disorder with variable degrees of insulin resistance and pancreatic *β*-cell dysfunction [1]. Overweight and obesity are major contributors to the development of insulin resistance and impaired glucose tolerance; when *β*-cells are no longer able to secrete enough insulin to overcome insulin resistance, impaired glucose tolerance progresses to type 2 diabetes [2, 3]. Abnormalities in other hormones such as reduced secretion of the incretin glucagon-like peptide 1 (GLP-1), hyperglucagonaemia, and raised concentrations of other counter-regulatory hormones also contribute to insulin resistance, reduced insulin secretion, and hyperglycaemia in type 2 diabetes [4–7]. Clinical-overt [8] and experimental [9] cortisol excess is associated with increased blood pressure and profound metabolic disturbances of intermediate metabolism resulting in abdominal obesity, insulin resistance, and low HDL-cholesterol levels, which can lead to diabetes. Therefore it has been suggested that subtle abnormalities in cortisol secretion and action are one of the missing links between insulin resistance and other features of the metabolic syndrome [10, 11]. The aim of this paper is to address the role of glucocorticoids on glucose homeostasis and to explain the relationships between hypercortisolism and diabetes. 

## 2. Glucocorticoids and Glucose Metabolism 

Glucocorticoid hormones are produced by the adrenal cortex under control of the hypothalamic-pituitary-adrenal (HPA) axis. They exert their function in different target tissues by binding two intracellular receptors: the glucocorticoid receptor and the mineralcorticoid receptor [12]. It is well known that the effects of glucocorticoids have a great variability between subjects, because of the different receptor sensitivity which is mostly genetically determined [13, 14]. Many other factors contribute to modulate the biological effects of glucocorticoids in target tissues; among them, the 11*β*-hydroxysteroid dehydrogenase (11*β*-HSD) enzymes, which interconvert cortisol to its inactive metabolite cortisone, are of particular interest: 11*β*-HSD type 1 (11*β*-HSD1) is mainly expressed in the liver and in the adipose tissue and amplifies local glucocorticoid action converting cortisone to cortisol, whereas 11*β*-HSD type 2 (11*β*-HSD2) is mainly expressed in the kidney and reduces glucocorticoid-induced effects by converting cortisol to the inactive cortisone [15] (see specific paragraph below). 

## 3. Hypercortisolism and Diabetes 

### 3.1. Endogenous Hypercortisolism (EH): Cushing's Syndrome/Disease

Cushing's syndrome (CS) is a clinical condition resulting from chronic exposure to glucocorticoid excess. EH can be either adrenocorticotropin hormone (ACTH) dependent (mostly due to pituitary adenomas, known as Cushing disease, CD) or ACTH independent (due to adrenal adenomas or hyperplasia) known as CS. Cortisol excess leads to a clinical phenotype that harbours all components of the metabolic syndrome as well as muscle weakness, hirsutism, increased bruisability, and psychological dysfunction [16–26]. Hypercortisolism is responsible for the occurrence of chronic metabolic complications, such as insulin resistance, diabetes mellitus, dyslipidemia, cardiovascular disease, and bone fragility [27], independently of the presence of obesity. 

Diabetes is considered to be a common complication of chronic exposure to glucocorticoid excess, and it is an important contributing factor to the morbidity and the mortality of the patients with EH [28]. The overall mortality in EH doubles the general population: in this clinical setting, diabetes, hypertension, and uncontrolled hypercortisolism have been shown to be important predictors of overall mortality [29–31]. Most of the deaths occur in patients with persistent hypercortisolism; moreover, patients who achieve the remission of the disease after many years of hypercortisolism have, however, a high risk of death [28]. 

### 3.2. Incidence of EH

It is widely accepted that CS is an uncommon disorder; however, its diagnosis is often delayed because of the difficulties in recognizing the clinical signs specific to this condition. In a Danish study, an incidence of two cases per one million persons per year was found [32], while a Spanish study showed an incidence of 2.5 cases per one million persons per year [29]. A recent large-scale retrospective survey carried out in New Zealand by Bolland showed that the prevalence of all forms of CS (the majority of these cases were of pituitary origin) was 79 cases per million, and the incidence was 1.8 per million per year [33]. 

### 3.3. Pathophysiology of Diabetes in EH

Insulin is produced and secreted by the pancreatic *β*-cells, which play a crucial role in the maintenance of glucose homeostasis; insulin secretion is stimulated by an increase in blood glucose levels and is modulated by hormonal and neuronal stimuli. The main intracellular signals in insulin secretion are ATP, Ca^2+^, cAMP, and phospholipid-derived signals. When blood glucose increases, it is transported into the *β*-cell by glucose transporters. Glucose metabolism increases ATP production in *β*-cell mitochondria, and the increase in the intracellular concentration of ATP closes the ATP-sensitive K^+^ channels. This causes depolarization of the *β*-cell membrane, opening voltage-dependent calcium channels, leading to intracellular Ca^2+^ influx. The rise in Ca^2+^ stimulates the exocytosis of insulin vesicles. Insulin secretion by the *β*-cell is greater when glucose is administered orally than with intravenous administration. This effect is triggered by the action of gastrointestinal hormones named incretins, such as glucagon-like peptide 1 (GLP-1) and glucose-dependent insulinotropic polypeptide, which act on insulin secretion by increasing cAMP levels in the *β*-cell. Insulin is secreted in a biphasic fashion: exposure of *β*-cell to an abrupt increase in glucose leads to insulin secretory-vesicle discharge. These insulin-filled vesicles are immediately available for exocytosis (first phase). The second phase is provided by granules of insulin mobilized from an intracellular pool. 

Glucocorticoids were shown to cause various degrees of *β*-cell dysfunction, reducing insulin sensitivity and impairing *β*-cell function [34], by acting through glucocorticoid receptors which are also expressed on pancreatic *β*-cells. Glucocorticoids may also impair the uptake and the metabolism of glucose in *β*-cells through genomic actions (i.e., modulation of gene expression by nuclear glucocorticoid receptor) which lead to a decrease in the efficacy of cytoplasmic Ca^2+^ on the exocytotic process of insulin secretory vehicles [35]. It was also recently reported that short-term exposure to glucocorticoids reduced the insulinotropic effects of GLP-1 [36]. The most important metabolic consequences of glucocorticoid excess occur during the postprandial period when these hormones exert anti-insulin effects in liver, skeletal muscle, and adipose tissue. Insulin favours the uptake and storage of glucose as glycogen in muscle and fat and reduces lipolysis by the inhibition of fatty acid release into the blood; insulin also inhibits hepatic gluconeogenesis and glycogenolysis. Glucocorticoid excess may affect all these biological activities leading to the development of insulin resistance. Moreover, glucocorticoids induce a postreceptor defect by decreasing key mediators of insulin action in peripheral tissues (insulin receptor substrate-1, phosphatidylinositol-3 kinase, and protein kinase B) [37]. These actions cause impaired glucose transporter translocation to the cell surface leading to a decrease in glucose uptake. The effects of glucocorticoid excess on hepatic gluconeogenesis have been also shown during fasting, as suggested by in vitro studies demonstrating a dexamethasone-induced activation of key enzymes involved in gluconeogenesis, such as phosphoenolpyruvate carboxykinase [38]. This effect may involve other proteins, such as liver X receptors, providing evidence for crosstalk between different metabolic pathways, such as glucose and cholesterol pathways, which could be affected by glucocorticoid excess [39]. Glucocorticoids exert their negative effects on insulin sensitivity also by modifying lipid and protein metabolism: they stimulate proteolysis, increasing aminoacid concentration which impairs different steps of insulin signalling, and increasing lipolysis which cause the elevation of free fatty acids that contribute to the impairment of glucose uptake and disposal [40]. Insulin resistance is also favoured by the abnormal distribution of body fat mass occurring in patients with CS, characterized by a specific increase of visceral adipose tissue. Finally, glucocorticoids may modulate the expression and the activity of adipokines, such as adiponectin, leptin, and apelin, which in turn may impair insulin sensitivity [41]. 

### 3.4. Incidence of Diabetes in EH and Diagnostic Tools

About half of CS patients have been shown to have alterations of glucose metabolism, and two thirds of these cases had diabetes, regardless of gender [42]. Diabetes occurs independently of the aetiology of glucocorticoid excess and does not seem to be correlated to disease duration. The close association between glucocorticoid excess and abnormalities in glucose metabolism is also demonstrated by the high unexpected prevalence of endogenous CS (up to 9%) in patients with diabetes mellitus, with difficult control of the disease and with coexistence of other features of glucocorticoid excess, such as central obesity and purple striae [43]. 

However, the available data do not support the screening for CS in diabetes clinics because of the high cost-effectiveness and the lack of specific tests for diagnosing CS, especially in patients with slightly elevated cortisol secretion and subclinical hypercortisolism [44]. Special consideration should be given to subclinical cortisol secretion in adrenal incidentalomas (see specific paragraph below). 

In patients with EH the measurement of fasting glucose may underestimate the real prevalence of glucose disorders, as suggested by the observation that more than one half of patients with EH and diabetes were shown to have normal fasting glucose [27]. This seems to be true also for patients taking exogenous glucocorticoids, in whom high glycemic values were shown to occur in the afternoon and in the evening [45]. The oral glucose tolerance test (OGTT) is therefore considered the diagnostic gold standard for identifying the impairment of glucose metabolism in EH [46], and for monitoring changes in glucose homeostasis. Measurement of glycated haemoglobin has been suggested for the diagnosis of diabetes in the general population and may be even more helpful in the clinical setting of EH because this parameter is an integrated measure of glucose homeostasis [47]. However, the use of glycated haemoglobin ≥6.5% for the diagnosis of diabetes is not yet of common practice because of problems in measuring this parameter and in interpreting the results in specific clinical conditions. Moreover, a high variability has been demonstrated in different ethnic groups in the sensitivity and specificity of glycated haemoglobin assays. Surrogate markers of peripheral insulin activity, such as homeostasis model assessment and whole-body insulin sensitivity indexes, could also be useful for the early identification of patients with insulin resistance before the development of overt diabetes [48]. 

### 3.5. Exogenous Hypercortisolism (ExH)

To date, the most frequent cause of hypercortisolism is the chronic therapy with glucocorticoids mainly prescribed for their anti-inflammatory effects through multiple pathways that promote the synthesis of anti-inflammatory proteins [49]. 

In addition to formulations intended to have systemic effects, topic formulations are widely used for specific clinical conditions and could also have systemic effects: intra-articular injections for arthritis, epidural injections for lumbar disk pain, eye drops for uveitis, nasal sprays for allergic rhinitis, inhalers for asthma, and topical ointments and creams for dermatological diseases. 

In glucocorticoid-treated patients, the odds ratio for development of new-onset diabetes mellitus has been reported to be from 1.36 to 2.31 [50]; however, the exact prevalence of diabetes in these patients has been reported only in few studies, showing that glucocorticoid-induced hyperglycaemia or diabetes is a common feature:

- nearly 9% of patients with rheumatoid arthritis have been shown to develop diabetes 2 years after starting glucocorticoid treatment [51];
- 42% of nondiabetic patients with primary renal disease treated with prednisolone 0.75 mg/kg/day were found to have 2-hour post-lunch plasma glucose concentrations higher than 200 mg/dL but normal fasting glucose levels [52]. The authors also reported that more than 50% of patients developed diabetes or impaired glucose tolerance 10 weeks after starting the treatment with prednisone (mean dose 10 mg/day); however, one of the limits of this study was the small number of patients;
- in a case-control study, the odds ratio (OR) of starting an oral hypoglycaemic agent or insulin in patients receiving a hydrocortisone-equivalent dose of 1 to 39 mg/day was 1.77; patients receiving higher doses of glucocorticoids have been shown to have higher ORs: 3.02 for 40 to 79 mg/day, 5.82 for 80 to 119 mg/day, and 10.34 for 120 mg/day or more [53].

Hyperglycaemia is a potential concern with both short-term (4 weeks or less) and long-term glucocorticoid treatments, such as in transplant recipients to prevent rejection or to treat graft-versus-host disease. However, there are no clear guidelines on the monitoring of blood glucose levels in patients undergoing long-term glucocorticoid treatment, and on the management of glucocorticoid-related diabetes, except for patients undergoing transplants: the international consensus guidelines published in 2003 suggest to check fasting plasma glucose level once a week for the first 4 weeks after transplantation, then at 3 and 6 months, and then once a year [54]. Though practical, this suggestion does not reflect the fact that glucocorticoids often do not affect fasting plasma glucose, especially if they are given once daily in the morning at doses of 30 mg or less of prednisone or its equivalent. The transplant guidelines do mention that an oral glucose tolerance test may be more sensitive, but this is often cumbersome to perform. Checking random postprandial plasma glucose levels could be helpful in this regard. 

The American Diabetes Association cutoff for diagnosing diabetes when using a random (i.e., nonfasting) plasma glucose level is 200 mg/dL or higher in patients with classic symptoms of hyperglycaemia such as polyuria and polydipsia [47]. If patients have risk factors for diabetes before receiving synthetic glucocorticoids (excess weight/obesity, family history of diabetes), fasting plasma glucose level ≥126 mg/dL or glycated haemoglobin ≥6.5% might suffice to diagnose diabetes (results should be confirmed on a separate day in the absence of unequivocal hyperglycaemia). 

### 3.6. Pathophysiology of Diabetes in ExH

Glucocorticoid-induced diabetes is similar to type 2 diabetes because glucocorticoids impair glucose metabolism mainly through increasing insulin resistance, which occurs in the liver with increased basal glucose production, and in the adipose and skeletal tissues with impaired glucose utilization: to better understand the mechanisms of glucocorticoid-induced hyperglycaemia, Pagano et al. [55] evaluated the effect of prednisolone administration for 7 days in healthy volunteers, showing 50% reduction in insulin sensitivity by using insulin clamp methods. These findings were supported by subsequent studies [56, 57]. Exogenous glucocorticoids could also interfere with the signalling pathways of various insulin secretagogues, but the exact mechanisms are still unknown. In vitro studies have been shown that glucocorticoids may reduce glucose uptake and oxidation and upregulate potassium-gated ion channels with impaired depolarization and decreased calcium influx (a stimulus for insulin granule release) [58–60]. Moreover, increased *β*-cell death after incubation with dexamethasone has also been shown [61].

### 3.7. Shared Features between Obesity, MetS, and Hypercortisolism

The metabolic syndrome (MetS) is a cluster of abnormalities that include central obesity, impaired glucose tolerance, hypertension, and dyslipidaemia [62–65]. Insulin resistance is one of the main defects which is shared between the individual components of the MetS although the strength of this correlation varies between, and even within, different populations [66]. 

Many studies have been shown a strong association between obesity, glucose intolerance, and diabetes: increasing body mass index (BMI) has been associated with increased incidence of impaired fasting glucose and diabetes in both sexes [67, 68]. However, the risk of developing diabetes has been shown to be higher in females: women with BMI of 24 to 24.9 kg/m^2^ had a risk of developing diabetes five times higher when compared to women with BMI of less than 22 kg/m^2^; the risk of developing diabetes in women with BMI greater than 31 kg/m^2^ and 35 kg/m^2^ was increased up to 40 times and 93 times, respectively [69]. 

Both insulin levels and BMI have been shown to be independent predictors of cardiovascular disease [70]. Similar results have been found in a study performed in male health professionals: the risk of diabetes was 42 times higher with BMI greater than 35 kg/m^2^ when compared to that of men with BMI less than 23 kg/m^2^ [71, 72]. The relationship between weight gain and diabetes appears to be relatively consistent among different ethnicities [73–75]. 

Glucocorticoids exert their effects also in the adipose tissue influencing its activity: they promote the differentiation and proliferation of human adipocytes acting by glucocorticoid receptors which are more abundant in visceral than in subcutaneous adipose tissue [76]; glucocorticoids redistribute adiposity from peripheral to central depots, as confirmed by studies showing a positive correlation between cortisol concentrations and intra-abdominal fat; finally, they increase size and number of fat cells and activate lipolysis and release of free fatty acids into the circulation [77]. 

Therefore, there is evidence that cortisol could play a role in determining the adiposity in MetS, even if there are conflicting results in the literature. Increases in urinary free cortisol excretion have been reported in patients with MetS [78, 79], whereas other studies did not confirm this finding, showing that the urinary cortisone/cortisol ratio in women with increased abdominal fat was higher if compared to those with peripheral fat distribution, suggesting an increase in the peripheral metabolism of cortisol [80]. Furthermore, some studies showed a positive correlation between cortisol and waist circumference [81–83], whilst other authors reported no relationship between these two parameters [83, 84]. 

However, several studies have shown an increased responsiveness of the HPA axis to different stimuli in patients with abdominal obesity: these stimuli included food intake [85, 86], low-dose tetracosactide [87], and CRH-arginine vasopressin [88, 89], leading to the conclusion that abdominal adiposity is associated with attenuated negative feedback in the HPA axis [90, 91] and with reduced diurnal variation in cortisol levels. Recently, Vicennati et al. [92] confirmed a positive relation between 24 h urinary free cortisol (UFC) values and waist circumference, independently of the BMI, in a cohort of overweight/obese women compared to normal weight subjects; a positive correlation between 24 h UFC levels and caloric intake, fat eating, and consumption of starchy foods, independently of BMI has also been shown. 

### 3.8. 11 Beta-Hydroxysteroid Dehydrogenase Type 1 and Type 2 Diabetes

The regulation of peripheral glucocorticoid levels is critical for the maintenance of homeostasis, playing a central role in pivotal physiological processes, such as stress responses, energy metabolism, electrolyte levels, blood pressure, immunity, cell proliferation and differentiation, and cognitive functions [93]. 

A major determinant of glucocorticoid peripheral action seems to be the expression of the 2 isoforms of the 11*β*-HSD, as mentioned before. 11*β*-HSD1 predominantly converts low-active cortisone to the more active cortisol. This enzyme is mainly expressed in the liver and in the adipose tissue, and its expression can be induced in fibroblasts, muscles, and other tissues (934). 11*β*-HSD2 converts cortisol to cortisone, and it has been found in tissues that express the mineralocorticoid receptor (especially the kidneys), allowing aldosterone to bind to this receptor [94]. 

Changes in the activity of these enzymes could lead to metabolic and hormonal alterations: increased 11*β*-HSD1 activity in visceral adipose tissue may generate increased cortisol levels within adipose tissue and liver, promoting the development of features of the MetS [95]. Transgenic mice overexpressing 11*β*-HSD1 selectively in the adipose tissue have been shown to faithfully recapitulate the stigmata of the MetS [96–98], whereas 11*β*-HSD1-KO mice treated with 11*β*-HSD1 inhibitors have been reported to be protected from cardiometabolic risks of the obesity [99]. 

Therefore, it has been suggested that MetS and central obesity may result from an increased availability of glucocorticoids specifically in the liver and in the adipose tissue, and this effect has been termed “Cushing's disease of the omentum” [100, 101]. Following this suggestion, few compounds with inhibitory activity of the 11*β*-HSD1 enzymes (INCB13739, MK-0916, MK-0736) have been tested in humans [102–105]; however, the major concern about these studies is the compensatory activation of the HPA axis as a result of the decrease of cortisol production in peripheral tissues due to the inhibition of the 11*β*-HSD1: the chronic hyperstimulation of the adrenal glands by ACTH might result in hyperproduction of adrenal androgens (dehydroepiandrosterone, dehydroepiandrosterone-sulphate, and Δ4-androstenedione) which may lead to clinical hyperandrogenism, and mineralocorticoid precursors which may cause salt retention and arterial hypertension [106]. More longitudinal data regarding the safety profile of these compounds are needed. 

### 3.9. Subclinical Cushing's Syndrome

Subclinical Cushing's syndrome (SCS) is defined as alterations of the hypothalamic-pituitary-adrenal (HPA) axis without the classic signs or symptoms specific to overt glucocorticoid excess [107, 108]. The clinical consequences of the long-term exposure to mild cortisol excess have yet to be defined, and the potential improvement of comorbidities after surgical treatment of SCS patients is still a matter of debate. 

It is well known that alterations of the glucose metabolism may occur in the presence of an excessive cortisol production, such as in overt CS, toward the complex net of mechanisms described above; however, studies on subclinical hypercortisolism available up to now are not able to answer the question if the same mechanisms are involved in the alterations of the glucose metabolism reported in these patients, and in the development of diabetes. 

The prevalence of SCS has been reported in many series of patients with adrenal masses discovered serendipitously, and it has been estimated in up to 30% of patients bearing an adrenal incidentaloma [109, 110]. In SCS patients, previous studies reported a variable prevalence of diabetes, ranging from 5% to 50% [111]. This wide variability could be mainly explained with 2 diagnostic biases: (i) because of the definition of SCS itself, the diagnosis of this condition in patients with adrenal incidentalomas is possible only using biochemical and hormonal criteria; moreover, the available guidelines lack a clear consensus on which are the best tests that should be used to define this condition [112, 113], leading to a nonhomogeneity in the classification of patients. (ii) The second bias is related to the diagnosis of alterations of the glucose metabolism, defined using fasting glucose and fasting insulin in some studies [114, 115], and using the OGTT in others [116, 117]. 

In a recent cross-sectional study that we performed on 348 patients with adrenal incidentalomas [118], we defined 4 groups of subjects with progressively increased patterns of subclinical cortisol hypersecretion, ranging from nonsecreting adenomas to intermediate phenotypes of cortisol hypersecretion, and to SCS. We used the 1 mg overnight dexamethasone suppression test as the primary criterion for the diagnosis of nonsecreting adenomas and SCS; basal ACTH and the UFC were used as adjunctive criteria to define the intermediate minor and intermediate major phenotypes. Diabetes was diagnosed according to the ADA guidelines. The prevalence of diabetes was similar in the nonsecreting patients and in the intermediate minor phenotype patients (15.2% and 18.3%), whereas it was higher in the intermediate major phenotype patients (32.7%), and even higher in the SCS patients (42.1%) (*P* = 0.004). Looking at these data it is possible to speculate that increasing patterns of subclinical hypercortisolism could indeed lead to alterations in the glucose metabolism; moreover, the prevalence of diabetes seems to increase according to the severity of the subclinical hypercortisolism. In this study we also showed an independent relationship between T2D and the SCS secreting pattern (with an independent contribution of age, which was higher in the SCS patients with respect to the nonsecreting patients). These data lead to the conclusion that the subclinical alterations of the HPA axis should be considered as a risk factor for diabetes. Although the cause-effects relationship between SCS and diabetes has still to be clarified, the beneficial effects of surgical treatment of SCS in order to improve or cure this disease are yet to be determined. Many studies reported an improvement of T2D after adrenalectomy: a prospective randomized study conducted on 45 SCS patients who were randomly selected for surgery or medical treatment showed an improvement of the T2D in 62.5% of patients after a mean followup of 8 years [119]. On the other hand, some studies did not confirm these findings [120]. However, many prospective studies have some biases, mainly related to the selection criteria for surgery, which lead to difficulties in interpreting the data and consequently in evaluating the efficacy of adrenalectomy in SCS patients with diabetes: patients undergoing adrenalectomy generally have an adrenal mass size >4 cm of diameter, or they have clinical complications of diabetes (such as high glycated haemoglobin, nephropathy, etc.) which are expected to improve after surgery. Large prospective and randomized studies are needed to evaluate the adequate treatment for SCS patients with diabetes. 

## 4. Summary and Conclusions 

Glucocorticoids exert deleterious effects on the glucose metabolism, leading to a wide range of alterations, from insulin-resistance to overt and complicated diabetes. The complex net of mechanisms that link hypercortisolism (endogenous or exogenous) to the development of these abnormalities is only partially understood. Understanding the mechanisms of glucocorticoid-induced glucose alterations could lead to the development of novel therapeutic anti-inflammatory drugs, with reduced impact on glucose metabolism. Finally, the link between hypercortisolism and metabolic syndrome deserves more interest: unravelling the open questions in this field could lead to a significant improvement in the treatment of obesity, diabetes, and its complications. 
